# Ecological interactions, host plant defenses, and control strategies in managing soybean looper, *Chrysodeixis includens* (Lepidoptera: Noctuidae)

**DOI:** 10.3389/finsc.2024.1480940

**Published:** 2024-12-12

**Authors:** Rahul Debnath, Justin George, Manish Gautam, Insha Shafi, Rupesh Kariyat, Gadi V. P. Reddy

**Affiliations:** ^1^ USDA-ARS Southern Insect Management Research Unit, Stoneville, MS, United States; ^2^ Department of Entomology and Plant Pathology, University of Arkansas, Fayetteville, AR, United States

**Keywords:** semiochemicals, IPM (Integrated Pest Management), insecticide resistance, tritrophic interaction, biological control

## Abstract

Soybean looper (SBL), *Chrysodeixis includens* (Walker 1858) (Lepidoptera: Noctuidae), is one of the most damaging insect pests of soybean, *Glycine max* (L.) Merr., in the mid-south region of the United States, and causes significant economic losses to cotton, sunflower, tomato, and tobacco crops in the United States, Brazil, and Argentina. Soybean production in the southern region accounted for 15.5% of the total production in the United States, and yield losses due to invertebrate pests were 5.8%, or 1.09 million metric ton, in 2022. As insecticide resistance of SBL continues to rise, the lack of alternate control strategies is a serious concern. Numerous studies have been reported on pest status, distribution, semiochemical-based attractant blends, pesticides and resistance mechanisms, host-plant resistance mechanisms, and molecular tools for controlling this pest in soybeans and other crops. However, there is no comprehensive review that summarizes and discusses these research on SBL and soybeans. The current management strategies for SBL remain heavily reliant on chemical insecticides and transgenic crops. In contrast, integrated pest management (IPM) strategies are needed to control the pest in an effective and environmentally friendly way. This review examines and synthesizes the literature on SBL as a significant pest of soybeans and other important crops, highlighting recent progress in ecological interactions, host plant defenses, and control strategies and identifying information gaps, thereby suggesting avenues for further research on this pest.

## 1 Introduction

*Glycine max* (L.) Merr. (Fabales: Fabaceae), or soybean, is the second most produced row crop in the United States (1). In 2023, the United States planted nearly 88.02 million acres and produced 4,417 bushels (million) of soybeans. *Chrysodeixis includens* (Walker, 1858) (Lepidoptera: Noctuidae) (commonly known as soybean looper, SBL) is a polyphagous pest. The caterpillars of SBL are voracious defoliators that cause significant damage to several crops, specifically soybeans, leading to devastating economic losses in the mid-southern United States. It feeds on almost 174 plant species belonging to 39 families (2), including common bean, sweet potato, tobacco, alfalfa, cotton, tomato, sunflower, okra, and morning glory. It can also adapt and consume other host plants (3). In the southern United States, where SBL is a major soybean pest, extensively dispersed throughout the Western Hemisphere (4), recently emerging as a major pest of economic importance in Brazil, specifically in cotton and soybean (5). In Argentina, it has also become a significant insect pest of soybean, which is the most important cultivated crop nationally (6). SBL is native to the Americas, specifically to North, Central, and South America. In North America, they occur predominantly in the southern and eastern United States, from Maine to Texas, with its presence extending as far west as California (3, 7). SBL’s distribution extends down to Argentina and Chile in the southern hemisphere (8). Also, they are reported in Australia and extend to the West Indies, and are reported from St. Helena Island in the South Atlantic Ocean (9, 10).

*Chrysodeixis includens* has developed resistance to multiple insecticide groups, including pyrethroids, carbamates, and organophosphates, throughout the southern United States (11). The widespread use of chemical insecticides against SBL is mainly associated with natural avoidance due to the larval tendency to stay underneath the leaf, which favors the development of resistance to insecticides (12). Concerns about field-evolved resistance to diamide-containing insecticides in SBL are currently prevalent in the southern United States due to dependence on a limited range of pesticide groups (13). *Chrysodeixis includens* is a widespread migratory pest, and adults migrate from Florida northward into Georgia and the Carolinas, as the temperature becomes more favorable for establishing their populations (14, 15). In agroecosystems where soybean and cotton are grown in close range, SBL outbreaks occur as cotton provides nutritious nectar for adults (16). A study by Moonga and Davis (17) reported that larvae raised on cotton have a greater net reproductive rate (*R*_0_), intrinsic rate of growth (*r*), and total number of progenies, compared with development on other crops under controlled conditions.

Recently, numerous research studies have been reported on SBL, including biology, ecology, host range, rearing techniques, sampling thresholds, olfaction, sex pheromones with attractant mixtures, chemical control methods, and IPM strategies to control SBL in soybean and other major row crops. Recent research has also led to the development of traps and monitoring methods using semiochemicals, novel cultivation practices, the appropriate timing for the introduction of biological control agents, the development of new strategies for host plant resistance using Cry proteins from *Bacillus thuringensis* (Bt), and molecular techniques in addition to IPM programs against SBL. However, a comprehensive review summarizing the research on integrated pest management of SBL has yet to be reported. This review mainly discusses recent advances in research on SBL, including the ecological interactions, host plant defenses, and different management methods, as well as future research directions in IPM.

## 2 Biology and life stages

*Chrysodeixis includens* is a lepidopteran polyphagous pest that causes significant economic damage to different crops (18). During the 2021 growing season, because of SBL infestation, total losses were 3,613 (bushels, in thousands), and targeted foliar insecticide application or replanting costs were $49,369 (in thousands) (DOI: doi.org/10.31274/cpn-20230511-0). There are many reports on the feeding damage caused by the larval stages of SBL on soybeans in different parts of North and South America and throughout Central America. The larvae form a characteristic feeding pattern on the underside of the leaves, moving from bottom to top and resembling a distinctive ‘window-like’ feeding pattern (19).

**Figure 1:**
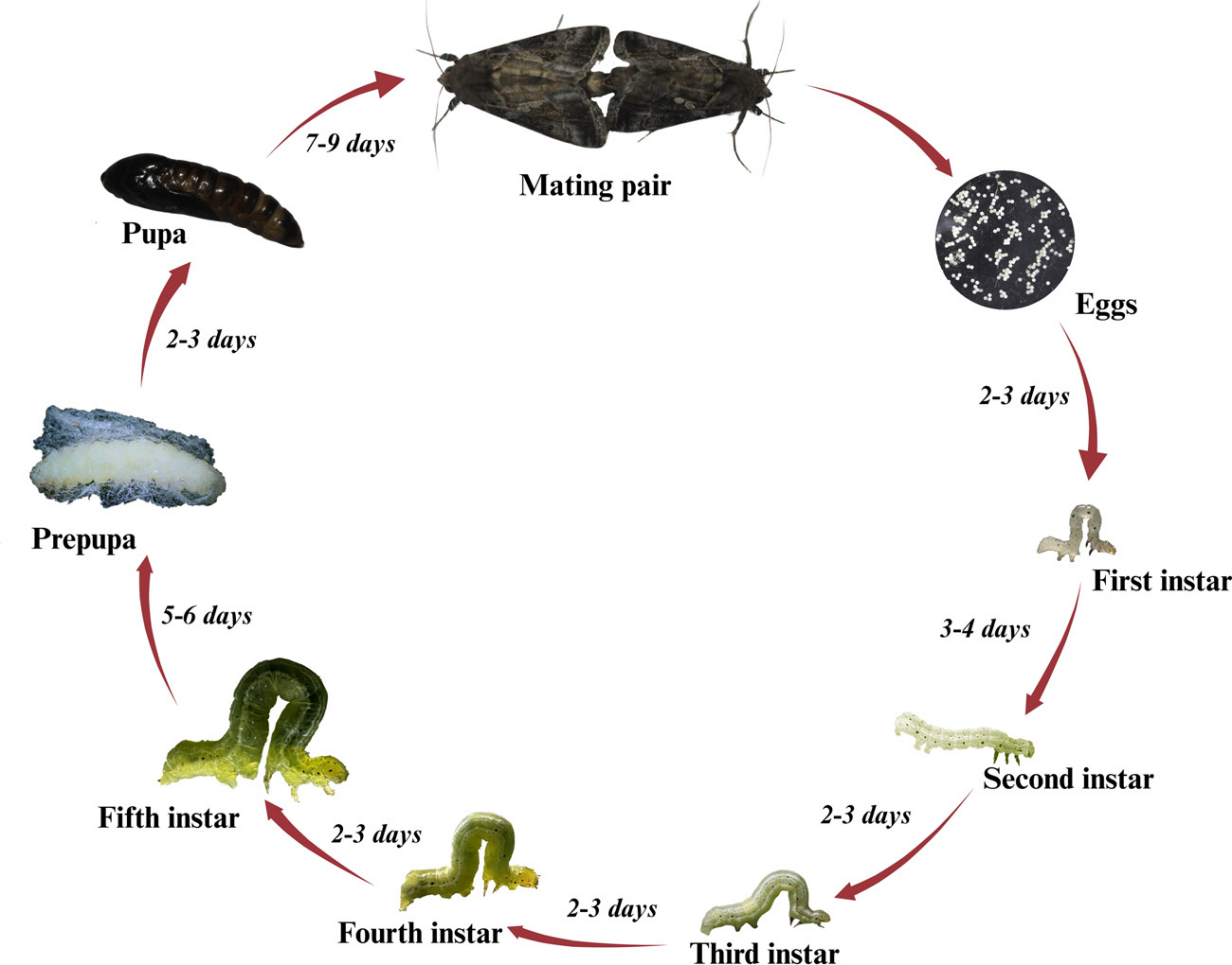
Life cycle stages of the soybean looper on soybeans, showing eggs, larval instars, pre-pupa, pupal stage, and adult mating pair.

Adult females deposit eggs singly in the lower half of the canopy on the underside of leaves at night and can lay around 600 eggs (20). The eggs are round and measure about 0.5 mm in diameter. After hatching, larvae typically transition through six different instars (21). Depending on various conditions, such as nutritional value of host plants and temperature, the first instar lasts 3-4 days, while most other instars last 2-3 days. The last instar is completed within 5-6 days (**Figure 1**). Larvae generally feed for 2-3 weeks before undergoing pupation (21). Larvae are light to dark green, with white stripes running across the dorsal and lateral parts of the body. The body of a larva is thicker and more prominent at the rear, tapers towards the head, and can grow up to 3 cm in length. The caterpillars of SBL can be distinguished from many similar-looking species like *Hypena scabra* (Fab, 1798) (Lepidoptera: Noctuidae) or green cloverworms, both species perform looping motions, while cloverworms possess three pairs of prolegs, whereas SBL have only two pairs of prolegs (19, 20). Pupation also takes place underneath the leaves; the pupa is covered by a silken cocoon and completed in 7-9 days. Pupae are about ~1.27 cm long and white to green color range (**Figure 1**) (20, 21).

The adult moth has a wingspan ranging from 2.54-3.81 cm and is brown to black. Forewings are darker than hindwings and possess silvery white spots, a distinguishable feature of the SBL (19). The longevity of female moths ranges from 13.5 to 14.8 days on different hosts (18). The larval developmental period varies between 29 days and 19 days on an artificial diet at 70 and 80°F, respectively, and larvae reared on soybeans in the field develop in 19 days, pupae develop in 7 days at 80°F (22). One generation of SBL is completed in less than a month in the southern United States, and in Louisiana, they can complete up to four generations a year.

## 3 Host plants, crop damage, promoting factors behind outbreaks, and a variety of defense mechanisms

### 3.1 Host plants and distributions

*Chrysodeixis includens* is a highly polyphagous pest that feeds on a variety of cultivated and weedy hosts, numbering about 174 plants belonging to 39 plant families, including cabbage (*Brassica oleracea* L.) (Brassicales: Brassicaceae), common bean (*Phaseolus vulgaris* L.) (Fabales: Fabaceae), lambs quarters (*Chenopodium album* L.) (Caryophyllales: Amaranthaceae), common morning glory (*Ipomoea purpurea* L.) (Solanales: Convolvulaceae), okra (*Abelmoschus esculentus* (L.) Moench) (Malvales: Malvaceae), Palmer amaranth (*Amaranthus palmeri* S. Wats) (Caryophyllales: Amaranthaceae), sweet potato (*Ipomoea batatas* (L.) Lam.) (Solanales: Convolvulaceae), tobacco (*Nicotiana tabacum* L.) (Solanales: Solanaceae), and tomato (*Solanum lycopersicum* L.) (Solanales: Solanaceae) (2, 23–25). A comparative study on the biology of SBL suggested that leaves of soybean, sunflower, and morning glory are favorable hosts for the development of the SBL. In contrast, the leaves of cotton (*Gossypium hirsutum* L.) (Malvales: Malvaceae), are less nutritionally adequate for overall development (18). In the absence of its preferred hosts, the SBL can adapt and feed on other plants (17) from families such as Alismataceae, Amaranthaceae, Apiaceae, Araceae, Araliaceae, Asteraceae, Poaceae, Solanaceae, Fabaceae, Euphorbiaceae, Malvaceae, Lamiaceae, Brassicaceae, Caryophyllaceae, Commelinaceae, and Convolvulaceae that are both cultivated and wild species (2).

SBL has been reported as a critical pest of soybeans in North America, and economic infestations in soybean occur north of Texas, Arkansas, Mississippi, Alabama, Georgia, and South Carolina (3, 26). Preferably, adult SBL lay their eggs in the lower part of the plants, but caterpillars mainly restrict themselves to the mid- and lower regions of plants (27, 28). The average density of SBL is highest during the complete bloom phase of soybean (28–30). They are better adapted to warmer regions. The highest occurrence of adults were observed during the reproductive stage of soybean. In the southern United States, SBL populations are most prevalent from August to September (31, 32). The permanent populations of SBL in the United States are thought to be restricted to the southern parts of Texas and Florida due to their inability to withstand winters in other regions, but the geographic range of the infestation is expanding annually (33). Each year, SBL migrate northward across several thousand miles in the United States, extending their territory during the same spring and summer growth season as a migratory pest (33, 34).

### 3.2 Crop damage

In southern states, SBL has four generations per year, with maximum populations observed in soybeans from late July to the end of August (28). *Chrysodeixis includens* is mainly a foliage feeder, and major damage comes from leaf feeding. Only immature pods serve as their food source, and they abstain from consuming pods with seeds developing in it. An individual SBL larva may consume 114-140 cm² of foliage, with 97% of the total intake happening during the late instars (62, 63). Early instars feed on the abaxial leaf surface and avoid the adaxial part, resulting in a clear window-like feeding pattern. Older larvae produce irregular holes and ragged margins by consuming the entire leaf from its margins. Large lateral leaf veins are often not fed on, giving the plant a ‘lace-like’ appearance (3, 5). Adult moths feed exclusively on nectar (20, 64).

Typically, soybeans do not become more resistant unless the same insect eats less leaf tissue at a later growth stages. Instead, the impact on yield from defoliation is not the same. The most vulnerable phases are the R3-R5 growth phase, R6 is less vulnerable, and R7 stage soybean are usually safe from major yield loss to defoliators like SBL. It was found that a high density of SBL eggs was observed in the full bloom stage (R2 growth stage) (28). Large populations of SBL are typically seen during the late season, increasing the risk of infestation for later maturing beans. Excessive defoliation can potentially impact yield indirectly by reducing the amount of photosynthetic energy the leaves produce for seed development. Because cotton nectar is a carbohydrate source that can significantly increase egg production by female moths, infestation rates tend to peak in the late season, especially in regions where cotton is also grown (16). In recent decades, very little research has been reported on SBL damage to soybeans in the southern United States. Analysis of agronomic practices and landscape factors can help predict yield losses in relation to pest abundance. Further research is needed to determine the distribution pattern, infestation rate, and intensity of damage caused by SBL in soybean fields.

### 3.3 Promoting factors behind soybean looper outbreaks

The proximity of cotton and soybean agroecosystems serves as the perfect combination for outbreaks of SBL because they often get a healthy supply of nectar from cotton (16). In recent years, *Helicoverpa zea (*Boddie) (Lepidoptera: Noctuidae) (podworm) and SBL have become the most destructive pest of soybean in southeastern states, responsible for approximately 24% insect management costs, and 12.9% of yield losses (65). It has been observed that SBL can often coexist with other lepidopterans that feed on leaves, such as *Anticarsia gemmatalis* (Hübner) (Lepidoptera: Noctuidae) (velvetbean caterpillars) and *H. scabra* (green cloverworms). If the combination of foliage feeding larvae cause damage up to 25%, then immediate control measures are justified (66).

Previously, SBL was considered as a secondary pest. Numerous outbreaks of SBL populations in soybean throughout South America over the past two decades have elevated this species as a major pest of soybeans (2). Musser et al. (65) reported that crop losses and management costs for SBL in the United States were estimated to be $56.8 million in 2019, which include majority of the range where SBL is considered a pest (67). In regions where both SBL and bean leaf beetle, *Cerotoma trifurcate* (Forster) (Coleoptera: Chrysomelidae) are present simultaneously, there is evidence that loopers attack the plants after the beetles (68, 69). Felton et al. (70) reported that *C. trifurcate* reduces the *H. zea* feeding and larval growth when they co-exist on soybean field.

Among different insect pests, the stink bug was the most economical pest in soybeans, followed by *H. zea* and SBL. Total insect management costs were $16.78 per acre, with estimated crop losses to insects at $15.36 per acre, making the total costs plus losses $32.13 per acre in 2022 (67). *Chrysodeixis includens* reproduce all year round as they do not diapause. They are known to overwinter in South Florida and South Texas in the continental United States. Since the 2013-2014 crop season, Bt soybean plants expressing the Cry1Ac protein have been approved for cultivation in Brazil (71, 72). The technology used in Bt soybeans allows for excellent management of SBL, and the trait appears to be a high-dose event in soybean. Selection of resistant populations remains challenging, especially considering how widespread this technology is in South America (73). Future research should investigate the phenological dynamic patterns and alternative host plants used by SBL, as this may help in the identification and management of potential population outbreaks, and is necessary for the development and recommendation of methods to minimize economic losses. Palma et al. (74) reported on the molecular characterization of SBL using inter simple sequence repeat (ISSR) markers. These are arbitrary markers generated by PCR that allow polymorphism detection in the regions of the inter-microsatellite loci consisting of di-, tri-, or tetra-nucleotide sequence repeats. Results of this study showed that high gene flow and low genetic differentiation were detected between different populations from different Brazilian regions, indicating recent re-colonization or migration patterns. A follow-up study by Silva et al. (75) described that the mean number of alleles per locus in three populations of SBL ranged from 2.33 for L3 to 14.67 for L6. Structural traits and F-statistics revealed low population structure and high levels of inbreeding. These suggests that future research on the ecology, demography, host dynamics, and gene flow of SBL will benefit from the use of SSR markers in this species. This information can be helpful in understanding the recent SBL population outbreaks in South America.

### 3.4 Defenses in soybean against soybean loopers

Soybean plants have developed a suite of resistance strategies and defensive mechanisms for protection from herbivore damage. These strategies include constitutive and induced defenses, which influence herbivore settling, feeding, oviposition, development, and reproduction (76). Constitutive defenses are physical and chemical traits expressed by plants regardless of herbivore presence, whereas induced defenses are activated only after herbivore attacks (77, 78). Both types of defenses are costly, as they divert nutrient resources away from vegetative and reproductive functions towards protective mechanisms (79).

#### 3.4.1 Constitutive defenses as trichomes

**Figure 2:**
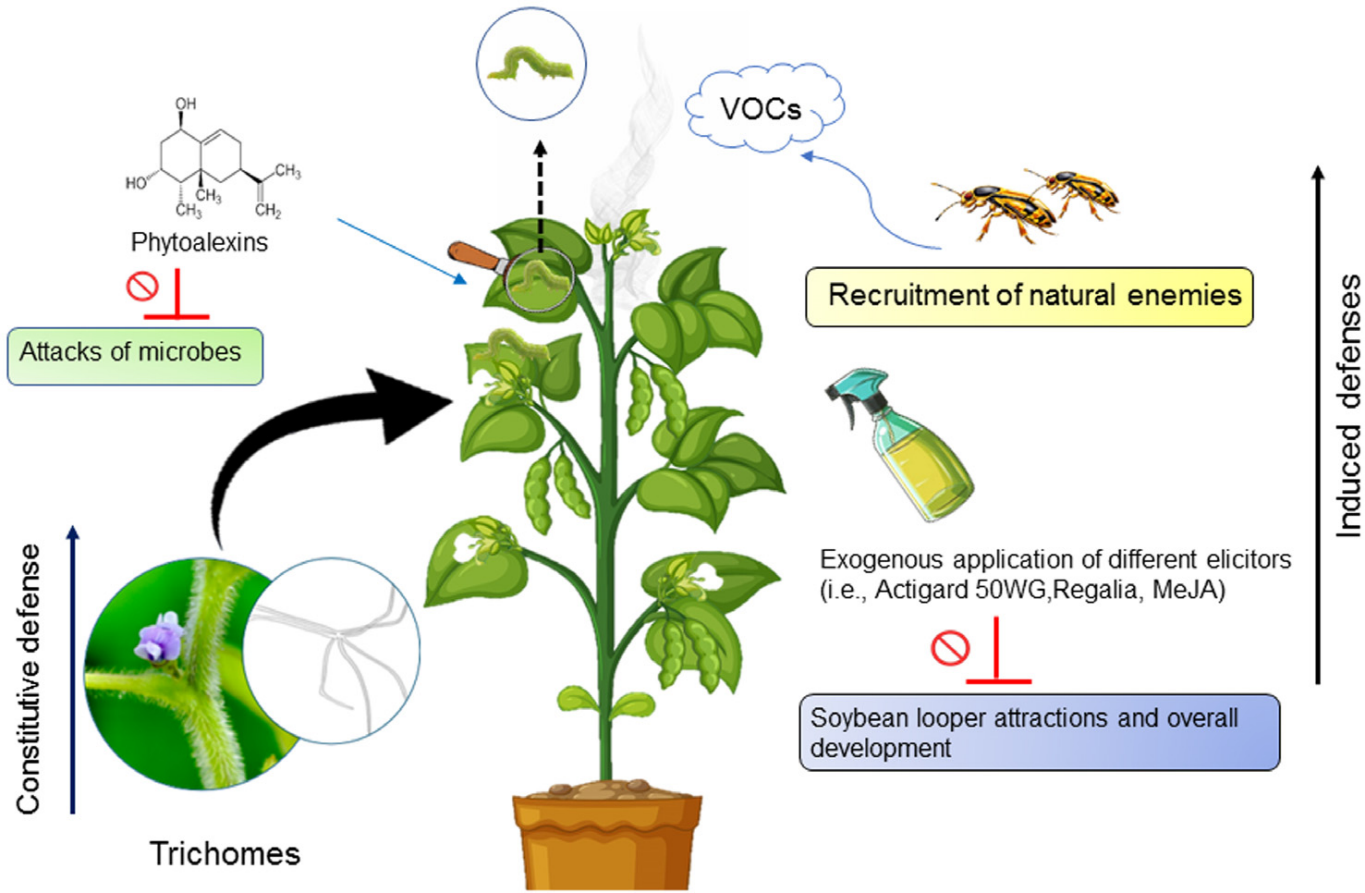
Schematic representation of the various defense mechanisms of soybean plants against infestation by soybean loopers.

Trichomes, hair-like structures present on nearly all above-ground parts of soybean plants, except for the hypocotyl and cotyledons, serve as a significant constitutive defense mechanism (80) (**Figure 2**). These structures can impede insect movement, feeding, and performance. Trichomes are well-documented for imparting host plant resistance in soybeans. Although specific studies on the impact of soybean trichomes on SBL are lacking, evidence from studies on related species suggests their effectiveness in herbivore defense. For example, research on the cabbage looper, *Trichoplusia ni* (Hubner, 1803) (Lepidoptera: Noctuidae), a close relative of the SBL, demonstrated that higher trichome density on soybean leaves reduced oviposition rates (81). This suggests a similar mechanism might apply to SBL due to their taxonomic relationship. Additional evidence from studies on *Pelargonium hortorum* (Bailey) (Geraniales: Geraniaceae) (garden geranium) which is mainly used as an ornamental plant, reveals that glandular trichomes produce an exudate highly toxic to SBL eggs and larvae. This exudate reduces egg hatch rates, increases mortality in early instar caterpillars, and induces vein-cutting behavior in final instar larvae (82). Krisnawati et al. (83) reported that soybean genotypes with maximum resistance to *Spodoptera litura* (Fab.) (Lepidoptera: Noctuidae) possess short and dense trichomes on both adaxial and abaxial leaf surfaces. These findings suggest that similar trichome characteristics could enhance resistance against SBL. While direct studies on soybean trichomes and SBL are limited, extensive research on related species and other pests offers compelling evidence of trichome-mediated plant defense. These findings underscore the potential importance of trichomes in soybean defense mechanisms against herbivory, suggesting a promising avenue for enhancing pest resistance in soybean cultivation.

#### 3.4.2 Induced defenses

Induced defenses in soybeans against herbivores involve a dynamic response to herbivore attacks, where the injury triggers various defense mechanisms (84–86). These responses may include the release of chemical compounds like phytoalexins and volatile organic compounds (VOCs), as well as changes in plant physiology and the recruitment of natural enemies (76, 87) (**Figure 2**). Smith (88) found that a soybean variety, (PI) 227687, resistant to SBL, triggers a cascade of induced defenses against herbivory, thereby resulting in heightened mortality and reduced fitness of the SBL larvae. Hart et al. (89) conducted a study investigating the impact of soybean phytoalexins on the feeding behavior of SBL and Mexican bean beetle. They observed that sixth-instars of SBL exhibited no preference between control and phytoalexin-rich tissues. Their findings indicate that while SBL may initially show no preference for phytoalexin-rich tissues, incorporating glyceollin into their diets could impact their survival rate. Lin and Kogan (90) evaluated induced resistance in soybean against SBL. Results showed that induced resistance slowed the development and growth of SBL. Besides this, Srinivas et al. (91) reported that SBL feeding induced a higher level of cross-resistance against bean leaf beetle feeding than vice versa, highlighting the complexity of induced defense mechanisms.

Similarly, elicitors, whether natural chemicals like jasmonic acid and salicylic acid or synthetic ones like methyl jasmonate and Actigard 50WG, play comparable roles to natural herbivores in triggering plant responses (92). These induced responses can potentially safeguard soybean plants by activating both direct and indirect resistance against pests at the appropriate developmental stages (93). Given the SBL’s resistance to numerous insecticides, induced host plant resistance emerges as a viable alternative control strategy. Chen et al. (87) demonstrated that the exogenous application of elicitors, including Actigard 50WG, Regalia, and methyl jasmonate (MeJA), negatively affected the developmental time, defoliation, and pupal weight of SBL, with MeJA exerting the most pronounced effects (**Figure 2**). These findings highlight the importance of induced defenses as a promising avenue for enhancing soybean resistance against herbivory. Further research into the mechanisms underlying these defenses could provide valuable insights for developing sustainable pest management strategies in soybean cultivation.

## 4 Behavior and ecology of *Chrysodeixis includens*

### 4.1 Semiochemicals

The advancement of mass trapping and mating disruption strategies, as well as their prospective application against SBL, depends on the accurate identification and optimization of their sex pheromones. Minimal studies have reported potential sex pheromones for SBL for attracting adults in soybean fields; hence, the discovery and optimization of such a sex pheromone for SBL are crucial (**Table 1**). Berger (97) first isolated, identified, and synthesized (Z)-7-dodecenyl acetate (Z7-12:Ac), the sex pheromone produced by the female cabbage looper. Follow-up studies showed that Z7-12:Ac was attractive to male moths of SBL in the laboratory as well as in the field (98). Mitchell (99) reported that both cabbage looper and SBL females belonging to the Noctuidae family produce this compound, likely making it the major component of the sex pheromone for both species. Although males of one moth species were attracted to the sex pheromone of conspecific females, they were not attracted to the pheromone of another species of female, which indicates the interaction is highly species-specific (100). Linn et al. (101) reported a five-component blend consisting of Z7-12:OAc as their most abundant compound, two other minor components: dodecyl acetate (12:OAc) and 11-dodecenyl acetate (11-12:OAc), and two different compounds added by them that are specific to SBL: Z-7-dodecenylpropionate (Z7-12: Prop) and Z-7-dodecenyl butanoate (Z7-12:But). This 5-component blend lure was found to give a higher level of response in SBL males than a single*-*component Z7-12Ac blend. Different studies simultaneously reported that the three main components of the sex pheromone blend of SBL consist of (Z)-7-dodecenyl acetate (Z7-12:Ac), (Z)-9-tetradecenyl acetate (Z9-14:Ac), and (Z)-11-hexadecenyl acetate (Z11-16:Ac) (68, 96, 97, 101). Results showed that the synthetic blend of these three components was highly attractive to male moths in field trials (68, 101).

**Table 1:** Semiochemicals eliciting soybean looper response/attraction.

Lure composition	Volatile type	Natural origin	References
Phenylacetaldehyde	Aldehydes	leaf/flower/fruit	Meagher (94)
Linalool + Phenylacetaldehyde	Terpene alcohol + Aldehydes	Flower	Meagher and Landolt (95)
Phenylacetaldehyde with 2-phenylethyl alcohol, methyl salicylate, dimethyl salicylate, benzaldehyde and benzyl alcohol	Aldehydes + primary alcohol + methyl ester aromatic aldehyde + aromatic alcohol	Flower	Stringer et al. (96)
(Z)-9-tetradecenyl acetate	Carboxylic ester	Sex pheromone	Flemming et al. (68)

Plants also defend themselves indirectly by releasing volatile organic compounds (VOCs) that may attract natural enemies of herbivorous insects to reduce the pest populations. Many agricultural crops, like soybeans, have been the subject of extensive research and reported on for induced plant resistance. Ramachandran and Norris (102) demonstrated that the responses of SBL and a parasitoid, *Microplitis demolitor* Wilkinson (Hymenoptera: Braconidae), to alcohols were similar; both were most sensitive to aldehydes and ketones and least sensitive to alcohols and hydrocarbons. Although, among the compounds tested, the least sensitivity to alcohols and hydrocarbons was proven by using electroantennogram studies. In two experimental setups, phenylacetaldehyde was used as a lure to capture Noctuidae family moths, including SBL, by using Unitraps (95). Stringer et al. (96) conducted an experiment to test traps for capturing SBL baited with synthetic lures derived from floral volatiles of the Canada thistle, *Cirsium arvense* (L.) (Asterales: Asteraceae), in combination with phenylacetaldehyde. They concluded that trap catches were higher when phenylacetaldehyde was combined with five prevalent volatiles present in *C. arvense* flowers. The abundance of female moths was twice that of males in trap capture. Further studies should explore whether these olfactory cues influence the behavior of SBL under different field conditions. Also, advances in understanding the behavioral ecology of SBL may help to create and apply efficient attract-and-kill approaches to minimize crop damage.

### 4.2 Trap characteristics associated with SBL capture

Herbivorous insects rely on multisensory integration of sensory cues to make decisions about the location of their host; in addition, visual cues can be crucial, especially for attraction at close range (103, 104). Therefore, optimizing the visual aspects of a trap could improve the effective capture of SBL under field conditions. Various studies have shown that SBL can be caught effectively by using different types of commercially available traps, including electric grid traps (98), pheromone-baited black light traps (99), wing traps (101), Hartstack traps (105), universal moth traps (95), and delta traps (28).

Among the different types of traps, delta traps have proven to be effective, reusable, and relatively cheap, with a replaceable sticky capture surface. Nevertheless, they are limited by the area of the sticky surface, which can become less sticky as moth capture increases and when dust or debris is blown into it (94, 96). Universal moth traps have the same favorable practical properties as delta traps, with the added advantage that they have a more extensive reservoir for the trapped moths, are less affected by wind-blown dust and debris, and the escape of captured moths is reduced when kill strips are used (28, 94).

### 4.3 Feeding performance of SBL

All instars of SBL primarily consume soybean leaves, but the third through fifth instars account for approximately 95% of total foliage consumption by the species (105, 106). Typically, early larvae prefer to feed on younger leaves, and late larval stages migrate to the upper canopy and feed indiscriminately on leaves (3, 107). Reid and Greene (63) reported that the consumption of leaves by the first three instars comprised only 3.3% of the total consumption; the final three instars performed the remaining 96.7% of consumption. Follow-up studies reported that the average total consumption for a larva was 113.82 cm^2^ as it matured through six instars (62, 108). Their findings indicate that larvae consumed an average of 0.13, 0.41, 1.99, 5.51, 12.18, and 93.48 cm^2^ of leaf tissue by one to six instars, respectively. Another similar study by Kogan and Cope (109) reported a total of 206.71 cm^2^ of leaf tissue consumed by individual larvae, and first through sixth instar larvae consumed on an average of 1.18, 3.36, 13.81, 26.35, 76.44, and 85.66 cm^2^, respectively, and 91.1% of leaf area was consumed solely by the last three instars. Further research involving the comparative analysis of feeding damaged by different SBL larval stages in field conditions could provide an estimation of the damage level performed by their population in a particular field condition.

## 5 Effects of external factors on soybean looper

### 5.1 Impact of abiotic factors

Abiotic factors such as drought, elevated temperature, and CO_2_ have pronounced effects on crops and, consequently, influence SBL biology, physiology, reproduction, and migratory behavior (110). Restricted water availability and severe drought have been found to alter the leaf water potential in soybean (109, 110), which could affect the growth and development of foliage-feeding herbivores like SBL. It has been well established that water-mediated changes in leaf water potential and chemical composition lead to induced defenses, such as trichomes (111). Higher trichome density in leaves serves dual purposes: the first is to reduce the loss of water via minimized transpiration, and the second is to defend against herbivores (112). Chen et al. (113) reported increased trichome density under the reduction of stem water potential due to water stress. This can potentially restrict the SBL movement, feeding and impact their growth upon ingestion in later stages.

Moreover, drought stress in soybeans has been found to be associated with a significant reduction in larval weights and prolonged larval developmental of SBL (35, 36, 114). In the case of SBL, according to Santos et al. (37), both excess and lack of rain raise larval mortality and alter their abundance. Similarly, Lambert and Heatherly (38) reported a substantial impact of water deficit on the larval development of SBL; larvae developing on irrigated soybeans were bigger and defoliated significantly greater proportions of leaves compared with larvae on water-deficit soybeans. Under simulated drought conditions, SBL performed poorly when feeding on stressed soybeans and showed non-preference behavior towards drought stressed soybeans (115). It has also been observed that water stress drives the SBL to look for a cooler and less humid canopy (114), which is uncommon in dryland farming systems. This suggests that, along with drought or water limitations in crops, temperature also plays a significant role in shaping host-insect interactions.

Like drought, temperature also affects growth and development of SBL throughout the larval and adult stages, but differentially. Mason and Mack (39) reported that female longevity decreased as temperature increased. Nevertheless, the temperature had a curvilinear relationship with the oviposition rate of SBL, which maxed at ~30°C and started decreasing beyond that temperature (39). On the other hand, Trichilo and Mack (107) observed a linear increase in the feeding rate of SBL, from 15.6°C to 32.2°C, suggesting that higher temperatures favored greater consumption of leaves by SBL. Therefore, it is evident that drought and temperature, two major stressors, prominently affect host location, development, mating, and dispersal of SBL.

Abiotic stressors also affect the trophic interactions of SBL and their predators. The nymphs and adult males of *Geocoris punctipes* (Say, 1832) (Hemiptera: Geocoridae) (bigeyed bugs), predators of SBL, were found to feed more on SBL eggs at higher temperatures (40), suggesting greater activity of predators with increasing temperature. Some studies have reported a relatively equal abundance of big-eyed bugs under drought and irrigated conditions (41). On the other hand, McPherson et al. (42) found that predators such as *Nabis* spp. and *Geocoris* spp. were more abundant under irrigated conditions. A comprehensive understanding of SBL interactions with different trophic levels under such abiotic stressors is clearly lacking and warrants further research.

Concurrently, it is evident that climate-driven events such as drought and fluctuations in temperature have consequences for host-insect interactions for most insect pests, with SBL being a migratory pest (30) and particularly sensitive to environmental changes. With a changing climate, looper overwintering range could expand, bringing them to more northern areas sooner, leading to an increase in pest status (30, 31). Recent rapid changes in climatic conditions, drought, and temperature, along with other abiotic factors such as elevated CO_2_, ozone level, greenhouse gases, and nutrient availability, will possibly undergo rapid dynamic fluctuation in the future. These abiotic factors could have significant cascading effects on SBL biology, mating, distribution, and migratory behavior, which are of great concern for several agricultural crops. The influence of climate, soil, and atmosphere on parasitoids can be significant, either directly or through plants. The degree of specialization of parasitoids significantly affects their response to varying abiotic conditions (43). Therefore, future studies should emphasize exploring the combined effects of several abiotic stresses on these traits to advance our knowledge of their impacts on SBL.

### 5.2 Consequences of long-term insecticide application

*Chrysodeixis includens* has developed resistance to almost every class of insecticide, including cyclodienes, DDT, organophosphates, carbamates, and pyrethroids, which makes it more challenging to control (7). Permethrin was initially effective at controlling loopers, but resistance was documented in 1987 (7). Reduction in neuronal and increased monooxygenase activity by cytochrome P450 are known to be the major mechanisms related to pyrethroid resistance in SBL and other lepidopteran pests (44, 116).

Control of SBL has become costly and challenging due to its tendency to acquire resistance to insecticides, mainly where cotton and soybeans are grown in close proximity. In particular, it was found that, in cotton-soybean agricultural ecosystems, permethrin resistance became more severe. This is likely due to the unintentional selection of SBL resulting from intensive pyrethroid treatment on cotton (45). Isbilir et al. (46), investigated the molecular characterization of the ryanodine receptor (RyR) and diamide resistance in SBL. In Puerto Rico, they observed considerable resistance to chlorantraniliprole. A study by Stacke et al. (12) found that the teflubenzuron resistance strain showed high cross-resistance to other chitin inhibitor insecticides, such as novaluron and lufenuron, but showed low cross-resistance to methoxyfenozide, flubendiamide, and indoxacarb.

Although the physiological mechanism underlying insecticide resistance in SBL is poorly understood, elevated levels of various metabolic enzymes were reported by Rose et al. (47), and Thomas and Boethel (44) discovered indications of target-site resistance. However, most of the studies conducted dealt with the resistance effect, so it is essential to gain knowledge about the genetic basis of resistance to insecticides in order to prevent or delay the effects of insecticide applications. In addition, studying the fitness costs associated with resistance can help to determine whether susceptibility can be restored without the use of insecticides.

## 6 Integrated management strategies

### 6.1 Sampling methodologies

Sweep nets and drop cloths are usually used to sample populations of SBL. But sampling biases associated with these methods are essential to consider when estimating populations of SBL. The drop cloth provides proximate population estimates for lepidopteran species, whereas the sweep net is a relative sampling method (48).

Different studies have examined the distinction between drop-cloth (also called shake-sheet) and sweep-net sampling by using either natural infestations or infesting known numbers of larvae in cages for the calculation of the recovery percentage (49, 50). Studebaker et al. (51) reported greater larval recovery by drop-cloth sampling, as soybean plants progressed through the reproductive growth stages, and recovery percentages for sweep net sampling were generally lower than those from drop-cloth sampling. Rudd and Jensen (52) conducted a comparison study between two sampling methods for SBL and reported that drop-cloth sampling was more effective than sweep-net sampling. They captured 12.4 SBL larvae (sample units are 1.82 row-m per sample) for every 10 larvae (per 25 sweeps) using sweep-net sampling. Marston et al. (50) converted the number of larvae collected by drop-cloth samples to whole-plant sampling and found that a higher percentage of larvae were recovered as the plants increased in growth stage and larvae increased in size.

### 6.2 Sampling thresholds

Economic thresholds for SBL in soybean fields are mainly based on visual estimations of defoliation percentage. Effective and efficient application of soybean insecticides can be achieved through the utilization of monitoring methods like sweep nets and pheromone traps. Flemming et al. (68) reported 2 types of trap (delta traps and bucket traps) and 3 commercially available lures (scentry lure, alpha scents) containing (Z)-7-dodecenyl acetate as the major active component of the lure used for SBL catch. The SBL go through four generations per year in Louisiana soybean fields. In Arkansas, the defoliation threshold is 40% in the vegetative stage and 25% in the reproductive stage, and defoliation in the reproductive stage mainly causes yield losses (53). Researchers in Arkansas, Mississippi, and Tennessee have identified different population thresholds for SBL larvae in sweep-net collections. Specifically, they found that 38 larvae per 25 sweeps are the threshold in vegetative stages, while 19 larvae per 25 sweeps are the threshold in reproductive stages (53, 54). Turnipseed (55) reported that the use of a shake-sheet for sampling SBL resulted in a higher number of larvae captured in comparison to the use of a sweep-net.

Defoliation by SBL can result in an overall reduction in yields for both irrigated and non-irrigated soybeans, while substantial yield reductions primarily occur during reproductive growth stages. Also, irrigated soybeans showed a more significant yield reduction of up to 50% than non-irrigated soybeans (54, 117). R3-R5 growth stages of soybeans had higher sensitivity to defoliation when compared with defoliation of the R6 stage (56), and one-time defoliation (16.5%) at the R3 growth stage (57). The ideal leaf area index (LAI) for maximum yield should be adjusted to the maturity group, stem maturity, and planting date. In the subtropical environment, soybean experiments and farms with a high level of technology exhibited a rise in yield (more than 4.5 metric tonnes ha^−1^), concurrent with an enlarged LAI (58). Major economic damage from SBL in the mid-south region is most likely during the period of reproductive growth of soybeans.

### 6.3 Cultural, mechanical, and physical control

The use of herbicide-resistant soybeans is a common practice among farmers in the United States, and were grown on 93% of the total soybean acreage in the United States in 2012 (118). Since 1996, glufosinate-resistant soybeans have been available to growers. In a previous review of IPM for soybean, Heinrich and Muniappan (119) reported that, for tropical farmers, cultural practices offer potential as IPM components because they are easy to implement, economical, practical, and safe for the environment.

Mascarenhas and Pitre (30) reported that an increased rate of oviposition by SBL in soybeans was found during the beginning pod (R3) developmental stage and the full pod (R4) developmental stage compared with the late vegetative (V4) and full bloom (R2) growth stages. Generally, planting dates are determined by climatic conditions, cultivars, and economic considerations. Early planting of early-maturing cultivars may be a means of cultural control for SBL in North American regions (120). Because, higher populations of SBL were observed in September than in June-July, early planting is suggested to avoid increased populations and late activity that results in massive damage (120, 121). As SBL migrates into soybean-growing regions, it takes time for populations to establish and cause severe damage levels. Also, a long time is required for soybeans to mature, and those that are planted later in the spring are at risk of severe damage. Additionally, row spacing has played an essential role in controlling SBL populations (122).

Throughout the Americas, SBL has showed sharp population peaks in different periods of the year (123, 124). As SBL is polyphagous, they maintain small populations surviving on alternate host plants throughout much of the year. In the presence of soybeans and favorable conditions, these populations can multiply rapidly (125). However, SBL preferred cotton and common beans as alternative hosts, which are also used in intercropping with soybeans (125). The use of these alternative hosts could, therefore, reveal changes in the population dynamics of SBL, further studies may clarify this inference.

### 6.4 Plant nutrition in relation to SBL infestation

Changes in plant nutritional composition could have an impact on metabolism and hormonal and signaling pathways, which may lead to changes in plant susceptibility and attractiveness to insects (126). In plants, potassium is crucial for the synthesis and transportation of primary metabolites and is responsible for the enhancement of specific nitrogenase activation and nitrogen fixation in the family Fabaceae like soybeans (127). Soybean plants can accumulate the highest amount of potassium at the R3 growth stage when soybeans begin podding (128). In a study by Chen et al. (128), growth and development of SBL were enhanced by optimal applications of potassium fertilizer. When soybean plants were fertilized with higher rates of potassium, growth of SBL was faster, and more leaf tissue was consumed, indicating that the foliage from potassium-treated plants is more conducive to SBL development. They found that, among a range of potassium fertilizer level (0, 33.6, 67.3, 100.9, 134.5, and 168.1 kg/ha of K_2_O), SBL consumed more leaf tissue and larval developmental time became shorter when feeding on foliage from the highest concentration treatments (128).

### 6.5 Host plant resistance strategies

Soybean plants that naturally resist SBL can be utilized for the management of SBL populations, offering an alternative approach to control with insecticides (132). Wille et al. (130) investigated the natural resistance of four soybean cultivars (BR 36, Benso 1RR, NA 5909 RG, and BMX Turbo RR) recommended for the southern Brazilian market to control SBL. The consumption of larvae was significantly higher on Benso 1 RR cultivars compared to other cultivars. The introduced genotypes PI 171451, PI 227687, and PI 229358 were mainly resilient to coleopteran insect pests (131). According to Boethel (132), these genotypes exhibited both antixenosis and antibiosis modes of plant resistance to major lepidopteran soybean pests. The use of PI 229358 was widespread in soybean genetic improvement initiatives in North Carolina, South Carolina, and Mississippi (133) decades ago. Smith and Gilaman (133) reported that when fed on PI 227687 foliage, larvae of SBL experienced significant mortality during both the early (3–7 days) and late (18–25 days) stages. Beach et al. (134) suggested that larvae of SBL that reared on resistant genotypes (GATIR 81-26, 81-306, 81-327, and PI 423968) consumed less foliage and had lower pupal weights than larvae reared on the susceptible genotype. Beach and Todd (135) suggested that the Georgia soybean variety, referred to as GatIR (81–296) displayed resistance to SBL and velvetbean caterpillar similar to that of the PI (229358) genotype.

For the last 25 years, farmers have benefited from the use of transgenic crops that produce *B. thuringiensis* (Bt) insecticidal proteins as effective pest management tools. The advantages of Bt technology in corn and cotton are endangered due to the evolution of resistance in several pests. Bt soybeans are not yet commercially available in United States. The sustainability of Bt crop resistance depends on the presence of non-Bt plants as a refuge against resistance (136). More Bt crops would result in inescapable selection pressure in *H. zea*, the major cotton pest and a corn pest for which soybean is a refuge.

### 6.6 Chemical control

*Chrysodeixis includens* have been controlled using various insecticide types, such as pyrethroids, carbamates, organophosphates, cyclodienes, DDT, and insect growth regulators (IGRs). Since the early 1960s, the southeastern United States has experienced insecticide resistance in the SBL population. Due to their capability to inhibit the enzyme acetylcholinesterase (AchE), organophosphates and carbamates were widely employed for managing SBL, but resistance was reported before 1970. From 1970 to 1972, the preference for managing SBL shifted towards methomyl, and its usage was recommended exclusively in Louisiana in 1974 (137). The mechanism of action for pyrethroids involves disrupting the electrical signaling in insects’ nervous systems by affecting the voltage-gated sodium channels within neuronal membranes. In the early 1980s, permethrin, a pyrethroid insecticide was introduced as a promising solution to SBL resistance to insecticides (138). During the early stages, the use of permethrin resulted in a significant level of effectiveness at one and four days post-treatment (18, 139). However, since 1987, resistance to SBL has been documented for pyrethroids in the United States (116, 140, 141). Stacke et al. (142) indicated that lambda-cyhalothrin-based pyrethroid resistance exists in SBL populations in Brazil. Populations of SBL resistant to cypermethrin and deltamethrin exhibit cross-resistance, potentially due to the similar molecular structures and binding sites of these pyrethroid insecticides (59, 60). *Chrysodeixis includens* in the central Delta region of Mississippi, as studied by Portillo et al. (61), exhibited enhanced resistance to pyrethroid insecticides. An observation was made that the resistance level between the SB91-1 and SB91-2 strains grew 2.52 times higher as the growing season advanced.

**Figure 3:**
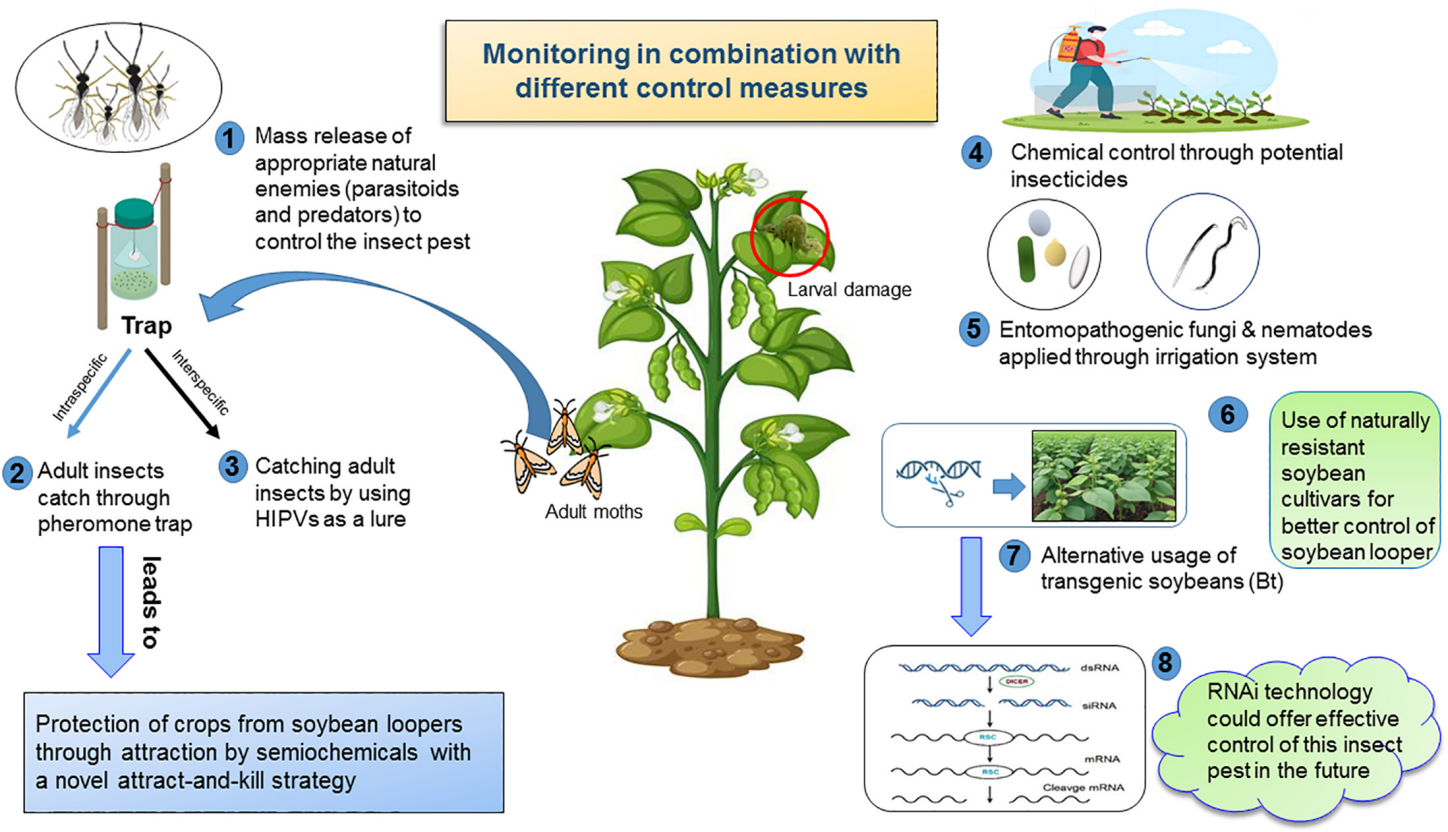
The schematic illustration depicts the recent advances in different management strategies to control soybean loopers in soybean fields that are IPM-compatible, making them outstanding for the frontier of ecologically sustainable control measures.

The first insect growth regulator (IGR) to enter the market was methoprene, which appeared in 1975. Thus, the applications of pyrethroid, carbamate, and organophosphate insecticides for management of SBL have mostly been replaced with different IGRs (i.e., novaluron, diflubenzuron, and methoxyfenozide). Among them, novaluron and diflubenzuron inhibit biosynthesis of chitin. Methoxyfenozide induces early molting by mimicking the molting hormone ecdysone (143). In 2013, ‘Intrepid Edge’ (a combination of methoxyfenozide and spinetoram) was approved for SBL management. In 2020, infestations of SBL were significantly reduced in plots that were treated with ‘Intrepid Edge’ compared to ‘Intrepid 2F’ and ‘Besiege’ (a combination of chlorantraniliprole and lambda-cyhalothrin) (144). Bonser et al. (129) first reported that the application of zein nanoparticles towards SBL and *Anticarsia gemmatalis* (velvetbean caterpillar) pests in soybeans resulted in the highest concentration of (+) ZNP being lethal to larvae of SBL (47%) and *A. gemmatalis* (95.6%). They recommended the potential use of (+) ZNP to control SBL. More investigation is needed to assess the impact and fine-tuning of ZNP application for managing SBL in field conditions. It is essential to devise insecticide application schedules, such as rotating or combining pesticides with particular modes of action, to hinder the development of resistance (**Figure 3**). Employing alternative IPM methods that are both effective and accessible to control SBL in field conditions may contribute to lowering insecticide applications and insecticide resistance in SBL in the southern United States.

### 6.7 Biological control

Parasitoids and predators serve as critical natural regulators for populations of SBL under field conditions. The use of appropriate natural predators to control larvae of SBL in field conditions would improve integrated pest management strategies (**Figure 3**). Burleigh (31) reported seven species of parasitoids that attacked larvae of SBL, and among them *Copidosoma truncatellum* (Dalman) (Hymenoptera: Encyrtidae) was the most prominent and abundant from mid-July until late August in Louisiana (**Table 2**). Three pathogens (*Nuclear polyhedrosis Virus (NPV)*, *Entomophthora gammae*, and *Nomuraea rileyi*) were identified in soybeans as well; among them, *NPV* was the most prevalent, infecting 63.7% of SBL larvae.

**Table 2:** Different parasitoids and fungus that commonly attack soybean looper larvae.

Family	Parasitoid/fungus	Parasitisation intensity and year	Infected host stage	References
Hymenoptera: Encyrtidae	*Copidosoma truncatellum* (Dalman)	15.9% in 1985	1st-6th instars	Diagle et al. (144)
Hymenoptera: Braconidae	*Cotesia marginiventris* (Cresson)	–	1st-4th instars	Diagle et al. (144), Beach and Todd (135)
	*Meteorus autographae* Ashmead	13.5% in 1984	1st-4th instars	Diagle et al. (144)
Hymenoptera: Icheumonidae	*Campoletis sonorensis* Cameron	–	larval stage	Burleigh (31)
	*Casinaria plusiae* Cavalier-Smith	–	larval stage	Burleigh (31)
Hymenoptera: Trichogrammatidae	*Trichogramma pretiosum* Riley	–	1st-4th instars (emerges during pre-pupal stage)	Harding JA (25)
Hymenoptera: Trachinidae	*Chaelophlepsis plathypenae* Sabrosky	–	3rd-5th instars	Diagle et al. (144)
	*Lespesia aleliae* (Riley)	–	4th-6th instars	Diagle et al. (144)
Entomophthorales	*Entomophthora gammae* Weiser	20.4% in1982-84	larval stage	Beach and Todd (135)
Clavicipitaceae	*Nomuraea rileyi* (Farlow) Samson	-	larval stage	Beach and Todd (135)

Pereira et al. (145) observed that larvae of SBL are frequently preyed upon by predatory ants, whose advanced hunting skills and foraging traits contribute to their significance as natural enemies. The main ant species responsible for predation on larvae were *Camponotus crassus* (Mayr) (Hymenoptera: Vespoidae), *Crematogaster evallans* (Forel) (Hymenoptera: Formicidae), *Pseudomyrmex gracilis* (Fabr.) (Hymenoptera: Formicidae), *Pseudomyrmex termitarius* (Smith) (Hymenoptera: Formicidae), and *Solenopsis saevissima* (Smith) (Hymenoptera: Formicidae). They also suggested that the suppression of these predators’ actions would lead to an increase in SBL populations. Beach and Todd (146) reported that parasitism by *C. truncatellum* influenced the feeding behavior and development of SBL larvae on a resistant and susceptible soybean genotypes. The parasitized larvae, when reared on the resistant genotype, had lower weights and resulted in fewer parasitoid adults in comparison to susceptible genotype, suggesting potential compatibility between the resistant soybean genotype and the parasitoid.

Richman et al. (147) observed that *Reduviolus roseipennis* (Reuter) (Hemiptera: Nabidae) and *Chrysopa* sp. larvae consumed numerous eggs of SBL, with an average of 28.6 and 19.13 eggs consumed daily, respectively. *Chracanthium inclusum* (Hentz, 1847) (Araneae: Cheiracanthiidae) was another egg predator that consumed 9 eggs per day. Nevertheless, further investigation is required to learn more about the impact of generalist predators on larvae of SBL in diverse field settings.

### 6.8 Microbial control

Insect control through microbial agents reduces chemical pesticide dependence and enhances environmental safety (**Figure 3**), though researchers need to conduct additional studies to improve effectiveness and affordability (148). The application of entomopathogens, such as nuclear polyhedrosis virus (NPVs) and entomopathogenic nematodes (EPNs), as microbial control agents (MCAs) against defoliating lepidopterans in soybean cultivation remains an avenue for further improvement (149). Additionally, using entomopathogenic fungi as microbial control agents has proven to be a crucial strategy for controlling insect pests. Entomopathogenic nematodes (EPNs) from the genera *Steinernema* and *Heterorhabditis* have proven effective against a range of insect pests both below and above the ground (150, 151). A recent study by Zhang et al. (152) has investigated EPNs against the SBL. This study tested ten EPN strains for their virulence against eggs, all five instars, and pupae of SBL in the laboratory. For SBL pupae, all strains, except *Heterorhabditis bacteriophora* (HP88), *Steinernema rarum* (17c+e), and *H. floridensis* (K22), had significantly higher mortality than controls. In field tests with the adjuvant 0.066% Southern Ag surfactant (SAg surfactant), *S. carpocapsae* had a huge impact on controlling fifth instars of SBL on soybean plants compared to *S. riobrave*. This study demonstrated that EPNs were adequate to control SBL, with SAg surfactant enhancing their efficacy.

In IPM, the use of entomopathogenic viruses offers an environmentally sound replacement for traditional chemical pesticides (153, 154). Baculoviruses belonging to the family Baculoviridae (nucleopolyhedrovirus and granulovirus) infect various insect orders, mainly different species of Lepidoptera (155, 156). The Baculoviridae family is comprised of large circular dsDNA (80-180 kbp) and bidirectionally oriented open reading frames (ORFs), which are distributed on both DNA strands and contain 37 genes (core genes) present in all Baculoviruses (157, 158). The *A. gemmatalis* nucleopolyhedrovirus (AgMNPV) of VBC is a well-known and widely recognized virus (148, 159), being utilized for soybean pest control in Brazil, Argentina, Paraguay, and Mexico. According to Alexandre et al. (160), SBL nucleopolyhedrovirus (ChinNPV), a highly virulent baculovirus, may be an appropriate bioinsecticide candidate for the targeted control of SBL. The ‘ChinNPV’ is a Group II alphabaculovirus, which is pathogenic and specific to SBL, previously identified under the genus *Pseudoplusia* (161). In 1972, researchers studied isolates of Psin-IA (I-A) under laboratory and field conditions to explore aspects related to dosage and temperature response. During field trials, the numbers of SBL larvae were significantly decreased in all plots where Psin-IA was applied (162). These viruses are promising selections for incorporation into management strategies owing to their high specificity and natural occurrence. Additionally, they are totally harmless to other microorganisms, natural enemies, pollinating bees, and host plants (148).

In a recent study by Harrison et al. (163), the novel alphabaculovirus identified as ChinNPV #1, derived from the SBL, was shown to control populations of SBL that had developed resistance to other SBL baculoviruses. The vast majority of the pathogens that infect soybean herbivore pest have not been utilized as MCAs at the farmer’s level. Further investigation is required to explore the effectiveness of this approach in the field and facilitate widespread implementation across different soybean growing areas in the southern United States.

## 7 Conclusion

Recent research on SBL has provided substantial progress with IPM strategies for control of this species, with a focus on soybeans in the southern United States and Brazil. Although significant advancements have been made, more research is necessary to establish sustainable methods for controlling SBL. Several generations of SBL occur during the year in the southern United States, and they overwinter in Florida and Texas. The complexity of dealing with SBL, especially in soybean agriculture, is heightened by the emergence of insecticide resistance in multiple cases. In the southern United States and Brazil, different IPM and crop management strategies have been assessed for dealing with SBL. SBL has a long history of developing resistance and is currently resistant to most classes of insecticides, like pyrethroids, carbamates, and organophosphates. The diacylhydrazide IGR, methoxyfenozide (Intrepid^®^ 2F, DowAgrosciences), was extensively employed to control infestations of SBL in the southern United States. While the excessive application of foliar insecticides for pest management can lead to resistance, the strategic use of this insecticide is recommended. Also, it is necessary to advance the research in order to develop strategies for managing insecticide resistance. Different studies have shown that resistance soybean genotype triggers a cascade of induced defenses against herbivory, thereby slowing the growth rate and feeding efficacy of SBL. Future investigations might offer significant knowledge for the progress and decision-making regarding resilient genetic strains.

Significant research has been conducted on the biology and ecology of SBL in the southern United States, but additional investigation is necessary. Additional research is essential to identify an efficient pheromone for detection of SBL. For large-scale cultivation in United States, further investigation is required to refine action thresholds for SBL before releasing Bt soybean technology to minimize the use of chemical insecticides. RNAi technology, which is based on exogenous dsRNA, is a promising option for next-generation insect pest control strategies. Studies employing RNAi techniques have been conducted on various insect orders. Multiple factors, such as dsRNA-degrading enzymes, cellular uptake efficiency, RNAi machinery expression, gene targets, dsRNA concentration, and insect feeding habits, can influence the efficiency of RNAi technology. Also, each insect poses unique challenges. So far, studies employing RNAi techniques to control SBL have not been reported (164). In summary, the continuous progress in SBL research has greatly enriched our knowledge about biology, ecology, population dynamics, host plant resistance, chemical control, and insecticide resistance. The current knowledge can be utilized for advancements in the integrated management of SBL.
